# Behavioural Risk Factors, Hypertension Knowledge, and Hypertension in Rural India

**DOI:** 10.1155/2020/8108202

**Published:** 2020-03-09

**Authors:** Eslavath Rajkumar, John Romate

**Affiliations:** Central University of Karnataka, Kalaburagi, India

## Abstract

Hypertension is an important health problem in India. The emergence of hypertension and other cardiovascular diseases are strongly related to various risk factors. Knowledge about hypertension and related risk factors is often stressed on their utility in prevention and management of the disease. Still, there is a poor understanding about associated behavioural risk factors of hypertension and importance of knowledge in adopting health-promoting behaviours and controlling hypertension among rural areas of India. This study aimed at assessing the association of behavioural risk factors with hypertension knowledge and hypertension among rural population. The present study focused on a south-western state of India from which a taluk with one of the lowest socioeconomic ratings was selected. A total of 263 participants were selected by using a multistage random sampling technique. Data were collected by in-person interview using behavioural risk factors questionnaire, hypertension knowledge questionnaire, and physical measurement. Data were analysed using descriptive statistics, chi square, Pearson correlation and binary logistic regression. Findings revealed that there is no significant relationship between risk factors index and knowledge of hypertension. It was also observed that factors such as smoking (OR = 0.29; CI: 090–0.961), fruit and vegetable consumption (OR = 1.32; CI: 1.01–1.74), body mass index (OR = 1.85; CI: 1.21–2.84), and age group (OR = 1.55; CI: 1.14–2.11) were significantly associated with the odds of hypertension. The factors such as smokeless tobacco use, alcohol consumption, physical activity, gender, education, and occupation were not associated with the odds of hypertension. Future research should focus on bringing down the associated risk factors to prevent and control hypertension.

## 1. Introduction

India, a lower middle income country with a population of more than 1 billion (rural population 68.84%), is undergoing a rapid epidemiologic transition characterized by an increased prevalence of noncommunicable diseases. Kumar et al. [1] report that in India, 3.78 million (40.4%) deaths in 1990 were due to chronic diseases while this figure is expected to reach 7.63 million (66.7%) by 2020. In 2012, heart disease and stroke were among the top three reasons that resulted in the decreased life span due to global premature mortality, and hypertension, one of the main risk factors for cardiovascular disease, contributes to 45% of heart diseases [2]. Hypertension is an important health problem in both urban and rural areas of India. It is estimated to account for 10.8% of all deaths and 4.6% of all disability-adjusted life years in India [3]. The emergence of hypertension and other cardiovascular diseases are strongly related to various risk factors such as aging of the populations, family history, socioeconomic changes that favor sedentary lifestyle, obesity, smoking, alcohol consumption, unhealthy dietary habits and stress. The prevalence of hypertension was higher among smokers (41.3%) as compared to nonsmokers (25.4%) and among obese (58%) compared to overweight (37.1%) and normal (25.3%) in a study conducted in Uttarakhand, India [4]. The cause of uncontrolled hypertension is multifactorial [5]. It is found to be associated with age, education subcategories, alcohol consumption status, and being overweight or obese [6]. The prevalence of hypertension was found to have increased with age among both genders, particularly after 45 years of age [7]. As age increased, the proportion of hypertensive patients in the different age groups also increased [8]. Ganesh et al. [9] found that factors such as being older, currently using alcohol, having less than 7 servings of fruits in a week, a moderate stress level and waist circumference more than 90 cm were associated with a higher prevalence rate of hypertension among a sample of male police personnel residing in urban Puducherry, India. Bartwal et al. [10] found that the prevalence of hypertension was 41.7% and there is a relationship of hypertension with increase in age, family history of hypertension, increase in salt intake, consuming mixed diet, increase in waist circumference, waist-hip ratio and body mass index. Similarly, Kokiwar et al. [11] found that while factors such as upper social class, sedentary physical activity, tobacco use, and diabetes had a significant association with hypertension, alcohol intake was not related to hypertension among rural communities of central India.

The World Health Organization [12] states that a significant percentage of noncommunicable diseases are preventable by addressing four main behavioural risk factors: physical inactivity, unhealthy diet, tobacco use, and alcohol consumption. Ard and Svetkey [13] reported that lifestyles such as physical activity and nutrition, play a vital role in preventing its long-term problems and controlling the hypertension. In order to improve their life condition, patients should have awareness about hypertension, the potential health risks associated with it and benefits of engaging in lifestyle modification [14]. Patients with hypertension need to have necessary skills and knowledge to take care of themselves, to define their condition, to understand and estimate potential risk factors and to appreciate how far lifelong medical control can help [15]. Low level of knowledge and awareness about blood pressure are also important factors for not adhering to antihypertensive drugs, which can lead to higher rates of uncontrolled blood pressure [16]. Patients who have good knowledge about their condition are more motivated to engage in blood pressure monitoring, which further resulted in medication adherence and blood pressure control [17].

By considering increment of hypertension in rural areas (men 12.6%; women 8.5%) [18], the importance of modifiable behavioural risk factors and hypertension knowledge in the prevalence and control of hypertension, the present study aimed to examine these constructs among rural India communities.

## 2. Method

### 2.1. Research Design

This study utilized a cross-sectional, survey to describe the association between behavioural risk factors, knowledge about hypertension, and hypertension.

### 2.2. Study Area and Sample

The present study focused on a south-western state of India. This community-based study was located in Jewargi taluk which ranks 174^th^ out of 175 taluks in the state of Karnataka on various socioeconomic indicators [19]. Based on 2011 census data, there were a total of 159 villages with a population of 2,96,000 in Jewargi taluk.

The multistage random sampling method was used to recruit 263 study participants. Initially, the researcher selected 3 villages randomly from Jewargi taluk by the lottery method.After selecting the villages, a unique number was given to each household in the three villages and another random draw was made to select sample households. A final lottery process was conducted to select the study participant among those in the household who were 18 years and above. Of 318 selected individuals, 263 consented to participate in the study, and the mean age of the participants is 44.3 years. The main reason given for nonparticipation was busy work schedule. Ethical clearance to conduct the study was obtained from the department and permission to conduct the study was obtained from the district health officer and taluk health officer.

## 3. Measures

### 3.1. Behavioural Risk Factor Index

The WHO Steps Tool (WHO) [20] was used to assess behavioural risk factors, namely, smoking, alcohol consumption, dietary behaviour, physical activity, and obesity. Obesity via a body mass index calculation was derived from the weight in kilograms divided by the height in meters squared (kg/m^2^). The participants were categorized as overweight (BMI greater than or equal to 25 to 29.9 kg/m^2^) and obese (BMI greater than or equal to 30 kg/m^2^). Each risk factor was coded 0/1 to represent presence of behaviour categorized by the Steps Tool. For example, being a current smoker, if physical activity was less than WHO guidelines, if fruit and/or vegetable consumption was lower than five servings/day, and being obese. Subsequently, a risk factor index was created by summing up across factors.

### 3.2. Hypertension Knowledge

The level of knowledge regarding hypertension was explored by asking questions about the reasons (high salt intake, family history, obesity, smoking, alcohol consumption, high fat diet, stress, lack of exercise, and unknown reasons), consequences (stroke, heart disease, kidney problem, eye problems, and diabetes), and preventive measures (reduce salt intake, reduce weight, reduce intake of fatty food, exercise regularly, start medicine, stop smoking, stop alcohol consumption and regular checkup) of high blood pressure and all the responses were recorded from the participants. Response options are dichotomous (yes/no) [21]. A single summed index score was calculated for knowledge of reasons, consequences, and preventive measures.

### 3.3. Hypertension

Blood pressure was measured three times with a minimum of 5-minute rest period in between each reading; participants were in a sitting position with the measurement on their right arm, taken over loose clothing using a portable digital blood pressure device. “Hypertension was defined as a systolic blood pressure 140 mm Hg or greater; or diastolic blood pressure 90 mm Hg or greater for average of the three readings; past history of diagnosis of hypertension; or receiving antihypertensive medication and captured in a dichotomous coded variable” [22].

### 3.4. Demographics

Six items were taken from the WHO Steps Tool [20] to assess age, gender, education, income, occupation, and marital status.

### 3.5. Data Collection

The written questionnaire was first translated from English to Kannada which is the most popular language in Karnataka. Three research assistants trained by the researcher conducted the in-person structured interviews after the consent forms obtained from the participants.

### 3.6. Data Analysis

All data were entered into SPSS 20^th^ version. Descriptive statistics were performed along with correlations and chi square to examine the relationships. Lastly, binary logistic regression was conducted to examine the contribution of risk factors and demographic details on hypertension status.

## 4. Results

Table 1 shows distribution of the participants according to hypertension status and gender. From the results, it was observed that majority of the hypertensive people were males (51.5%) compared to females (48.4%). When the participants were compared according to the educational qualification, majority of the hypertensive people had no formal schooling (67.1%) followed by less than primary school (12.5%) education. When the participants were compared according to occupation, majority of the participants were daily-wage labourers (28.1%), followed by unemployed and unable to work (26.5%), and self-employed (20.3%) people, respectively. Majority of the hypertensive people were married (78.1%). While comparing the participants based on the age group, majority of the hypertensive people were found to be of the age group 46 and above (64%).

Furthermore, genderwise distribution showed that majority of the participants belonged to the age group of 46 and above (males, 48.9% and females, 37.8%). The majority of the study subjects in both groups had no formal education (55% of males and 78% of females). When the participants were compared according to the occupation, most of them belonged to the category of daily-wage labourers (32.5% of males and 42.1% of females). Majority of the participants in both the groups were married (male, 85.4% and female, 80.7%).

Table 2 shows genderwise distribution of the subjects with respect to different risk factors. From the results, it was found that there was a difference between males (26.1%) and females (0%) with regard to smoking (*p* < 0.01). Males were found to smoke more than females. With regard to smokeless tobacco use, there was no difference found between males (39.1%) and females (37.1%). With regard to alcohol consumption, males were found to consume more alcohol than females (males, 33.3% and females, 0.7%),(*p* < 0.01). Similarly, there was a difference found between males and females with regard to fruit and vegetable consumption: males were found to consume more amount of fruits and vegetables than females (4% and 0.8%, respectively). There was a difference found between males (83, 67.4%) and females (94, 67.1%) with respect to physical activity, 18.6% and 17.1% with regard to overweight, and 5.6% and 5.3% with regard to obesity, respectively, for males and females.

Table 3 shows distribution of risk factors between hypertensive and nonhypertensive people. From the results, it was found that there was no statistical significant difference between hypertensive (7.8%) and nonhypertensive (13.5%) people with regard to smoking, but majority of the smokers were observed to be in the nonhypertensive group. With regard to smokeless tobacco use, there was no significant difference found between hypertensive (42.1%) and nonhypertensive (36.6%) people, but majority of the smokeless tobacco users were found in the hypertensive group. There was no difference found between hypertensive (17.1%) and nonhypertensive (15.5%) people with regard to alcohol consumption, but majority of the alcohol consumers were found in the nonhypertensive group. Similarly, there was no significant difference found between hypertensive (4.6%) and nonhypertensive (1.5%) people with regard to fruit and vegetable consumption. There was no significant difference found between hypertensive (59.3%) and nonhypertensive (69.8%) people with regard to physical activity; 28.1% hypertensive and 14.5% nonhypertensive with regard to overweight; and 6% hypertensive and 5% nonhypertensive with regard to obesity. Majority of the overweight and obese people were found in the hypertensive group.

From Table 4, it was observed that there was no significant relationship of risk factor index with reasons of hypertension knowledge index (*r* = −0.08, *p* = 0.17), consequences of hypertension knowledge index (*r* = 0.04, *p* = 0.50), and knowledge of preventive measures index, respectively (*r* = −0.11, *p* = 0.06).

In Table 5, binary logistic regression analysis findings showed that smoking behaviour (OR = 0.29; CI: 090–0.961) was significantly associated with the odds of hypertension. Although in reality, there is an association between smoking and hypertension, in the present study, as the value of odds ratio was lesser, the results have shown smokers to have lower risk of hypertension; it could be attributed to the less number of samples in the study. Furthermore, fruit and vegetable consumption (OR = 1.32; CI: 1.01–1.74) was significantly associated with the odds of hypertension. In the present study, the odds of hypertension was greater than one, but very few people in the study who have hypertension consumed more than five servings of fruits and vegetables. In addition to these, body mass index (OR = 1.85; CI: 1.212–2.84) and age group (OR = 1.55; CI: 1.14–2.11) were significantly associated with the odds of hypertension. The factors, including smokeless tobacco use, alcohol consumption, physical activity, gender, education, and occupation, did not predict the odds of hypertension.

## 5. Discussion

Hypertension is an important health problem in both urban and rural areas of India. The present study found that the prevalence of hypertension was 24% which is similar to the WHO findings [12] which stated that 23.10% of men and 22.60% of women over 25 years suffer from hypertension.

From the findings, it is observed that there is no significant association between risk factors index and knowledge about reasons, consequences, and preventive measures of hypertension. From the findings, it could be inferred that knowledge alone was not sufficient either to control health risk factors or to adopt health-promoting behaviours. Even knowledge is an important determinant to change behaviour or lifestyle; there might have been other factors which played an important role in transferring knowledge to practice. Al Deagi et al. [23] found that sufficient awareness about diabetes is associated with poor adherence to recommendations, and gaining knowledge related to diabetes was not sufficient to increase adherence with diabetes treatment [24]. Klepac [25] found that individuals did not undertake a health-related behaviour if they lacked at least a minimum amount of health motivation and knowledge. It is established that higher level of knowledge about the disease leads to better self-care and compliance to treatment or management. There was, however, an inconsistency between knowledge of the disease and complying with drug therapy and disease management. Thus, there was a discrepancy between knowledge and practice, which meant that despite knowing what needs to be done, participants did not act accordingly. This is because while the knowledge has a rational element, the adherence involved a lot of variables such as emotional, social, biological, and cultural factors [26]. Aubert et al. [27] reported that most people had enough knowledge but only a few were motivated and wished and attempted to have change, and very few had translated it into practice, whereby actively engaging in a new behaviour. There are various reasons for having low outcome expectation on chronic disease control and reluctance in adopting healthy lifestyles. Primarily, people may not be serious about the nature and effects of hypertension because of its silent progression, chronic nature, and late impact on health outcomes. Secondly, adopting lifestyle in a society is framed by the common attitudes, beliefs and social conditions. Thirdly, indulging in immediate pleasurable behaviours like enjoying food filled with fat and salt, avoiding physical exercise, and smoking deterred the adoption of healthy behaviours [28]. Finally, individuals perceived that they either lacked the needed skills to follow the routines of a healthy lifestyle or that they were unable to afford them. Nourjah et al. [29], in their discussion on health behaviours of blue and white collared employees, reported that the differences in individuals' beliefs, values, and their social norms could not be ignored in adopting healthy behaviours. Gayathri et al. [30] also reported that there is a positive relationship between health locus of control and engaging positive health behaviours. As a conclusive note, it could be inferred from the results that knowledge was not only the determinant that makes people to adopt health-promoting behaviour, but there are many other factors which need to be taken into account when talking about the prevention of risk factors.

Further findings showed that behavioural risk factors such as smoking, fruit and vegetable consumption, body mass index and age group significantly predicted the odds of hypertension. These findings were similar to those reported by Awino et al. [31], Laxmaiah et al. [32], and Mahmood et al. [33] found that age, education, obesity, smoking, and alcohol consumption were predictors of hypertension; similarly, Ganesh et al. [9] also found that factors such as higher age group, current use of alcohol, less than 7 servings of fruits in a week, moderate stress level, and waist circumference more than 90 cm were associated with higher prevalence of hypertension.

## 6. Conclusion

Prevalence of hypertension and risk factors is high among the study population. While knowledge was an important determinant to change behaviour or lifestyle, in the present study, no correlation was found between risk factor index and knowledge about hypertension. Further findings showed that behavioural risk factors, including smoking, fruit and vegetable consumption, body mass index, and age group, significantly predicted the odds of hypertension. The factors such as smokeless tobacco use, alcohol consumption, physical activity, gender, education and occupation were not associated with the odds of hypertension. Future research should focus on bringing down the associated risk factors and prevent hypertension.

### 6.1. Limitations and Future Directions of the Study

As the present study was conducted in a rural population, its findings cannot be generalized outside the rural sample. The researcher has collected the data from all the participants whose age group was 18 and above, but the study did not emphasize by taking specific age group into the consideration. Since the present study was a cross-sectional study, cause and effect relationship could not be established. So, in the future, the study should be extended to different parts of the country by stratifying the age groups.

### 6.2. What Is Known about This Topic?

1. In India, prevalence of hypertension is increasing among the younger age individuals; 10.8% of all deaths and 4.6% of all disability-adjusted life years in India result from hypertension.
2. The emergence of hypertension and other cardiovascular diseases is strongly related to various risk factors. Still, there is poor understanding about associated behavioural risk factors of hypertension and importance of knowledge in adopting health-promoting behaviours and controlling hypertension among rural areas of India.

### 6.3. What This Study Adds?

1. There is no significant relationship of risk factor index with knowledge of hypertension.
2. The factors such as smoking, fruit and vegetable consumption, body mass index, and age group significantly predicted the odds of hypertension.
3. The factors, including smokeless tobacco use, alcohol consumption, physical activity, gender, education, and occupation, did not predict the odds of hypertension.

## Figures and Tables

**Table 1:** Distribution of demographic information between hypertensive status and gender.

Demographic variables	Hypertension status			Gender		
	Not hypertensive 199	Hypertensive (64)	Total	Males	Females	Total
*Gender*						
Male	90(45.2)	33 (51.5)	123 (46.7)	123 (46.7)	140 (53.2)	263 (100%)
Female	109 (54.7)	31 (48.4)	140 (53.2)			

*Education*						
No formal schooling	122 (61.3)	43 (67.1)	165 (62.7)	55 (44.7)	110 (78.6)	165 (62.7)
Less than primary school	33 (16.5)	8 (12.5)	41 (15.5)	29 (23.6)	12 (8.6)	41 (15.6)
Primary school completed	13 (6.5)	3 (4.6)	16 (6)	7 (5.7)	9 (6.4)	16 (6.1)
Secondary school completed	1 (0.5)	1 (1.5)	2 (0.7)	2 (1.6)	0	2 (.8)
High school completed	12 (60.3)	6 (9.3)	18 (6.8)	14 (11.4)	4 (2.9)	18 (6.8)
College/graduation completed	17 (8.5)	3 (4.6)	20 (7.6)	16 (13.0)	4 (2.9)	20 (7.6)
Postgraduation and above	1 (0.5)	0 (0)	1 (0.3)	0	1 (0.7)	1 (.4)

*Occupation*						
Government employee	2 (1)	0 (0)	2 (0.7)	2 (1.6)	0	2 (.8)
Non-government employment	3 (1.5)	4 (6.2)	7 (2.6)	5 (4.1)	2 (1.4)	7 (2.7)
Self-employed	44 (22.1)	13 (20.3)	57 (21.6)	44 (35.8)	13 (9.3)	57 (21.7)
Nonpaid	6 (3)	2 (3.1)	8 (3)	3 (2.4)	5 (3.6)	8 (3.0)
Student	5 (5.1)	1 (1.5)	6 (2.2)	4 (3.3)	2 (1.4)	6 (2.3)
Home maker	30 (15)	7 (10.9)	37 (14)	0	37 (26.4)	37 (14.1)
Unemployed, able to work	4 (0.2)	2 (3.1)	6 (2.2)	4 (3.3)	2 (1.4)	6 (2.3)
Unemployed, unable to work	24 (12)	17 (26.5)	41 (15.5)	21 (17.1)	20 (14.3)	41 (15.6)
Daily wage labourer	81 (40.7)	18 (28.1)	99 (37.6)	40 (32.5)	59 (42.1)	99 (37.6)

*Marital status*						
Never married	18 (9)	4 (6.2)	22 (8.3)	14 (11.8)	8 (5.7)	22 (8.4)
Currently married	168 (84.4)	50 (78.1)	218 (82.8)	105 (85.4)	113 (80.7)	218 (82.9)
Separated	2 (1)	0 (0)	2 (0.7)	1 (.8)	1 (.7)	2 (.8)
Widowed	11 (5.5)	10 (15.6)	21 (7.9)	3 (2.4)	18 (12.9)	21 (8.0)

*Age in years*						
Age group 18–30	62 (31.1)	8 (12.5)	70 (26.6)	25 (20.3)	45 (32.1)	70 (26.6)
Age group 31–45	65 (32.6)	15 (23.4)	80 (30.4)	38 (30.9)	42 (30)	80 (30.4)
Age group 46 and above	72 (36.1)	41 (64)	123 (46.7)	60 (48.9)	53 (37.8)	113 (43)

**Table 2:** Chi square shows distribution of risk factors in association with gender.

Risk factors	Males (123)	Females (140)	Total	df	*p*
*Smoking*					
Current smokers	32 (26.1)	0 (0)	32 (12.1)	1	0.01
Nonsmokers	91 (73.9)	140 (100)	231 (87.8)		

*Smokeless tobacco*					
Smokeless tobacco users	48 (39.1)	52 (37.1)	100 (38.1)	1	0.75
Smokeless tobacco nonusers	75(60.9)	88 (62.8)	163(61.9)		

*Alcohol consumption*					
Current alcohol consumers	41 (33.3)	1 (0.7)	42 (15.9)	1	0.01
Nonconsumers	82 (66.6)	139 (99.3)	221 (84.3)		

*Fruit and vegetable consumption*					
>5 servings of fruit and vegetable consumption/day	5(4)	1 (0.8)	6 (2.2)	1	0.07
<5 servings of fruit and vegetable consumption/day	118 (95.9)	139 (99.2)	257 (97.7)		

*Physical activity*					
Less than the recommended level by the WHO (600 MET-minutes/week)	40 (32.5)	46 (32.8)	86 (32.6)	1	0.95
More than the recommended level by the WHO (600 MET-minutes/week)	83 (67.4)	94 (67.1)	177 (67.3)		

*BMI*					
Underweight	20 (16.2)	29 (20.7)	49 (18.6)	3	0.82
Normal	73 (59.3)	80 (57.1)	153 (58.1)		
Overweight	23 (18.6)	24 (17.1)	47 (17.8)		
Obese	7 (5.6)	7 (5)	14 (5.3)		

**Table 3:** Chi square shows distribution of risk factors in association with hypertension status.

Risk factors	Hypertensive, 64 (*n* %)	Nonhypertensive, 199 (*n* %)	Total	df	*p*
*Smoking*					
Current smokers	5 (7.8)	27 (13.5)	32 (12.1)	1	0.22
Nonsmokers	59 (92.1)	172 (86.4)	231 (88.4)		

*Smokeless tobacco*					
Smokeless tobacco users	27 (42.1)	73 (36.6)	100 (38)	1	0.43
Smokeless tobacco nonusers	37 (57.8)	126 (63.3)	163 (61.9)		

*Alcohol consumption*					
Current alcohol consumers	11 (17.1)	31 (15.5)	42 (15.9)	1	0.76
Nonconsumers	53 (82.8)	168 (84.4)	220 (83.6)		

*Fruit and vegetable consumption*					
>5 servings of fruit and vegetable consumption/day	3 (4.6)	3 (1.5)	6 (2.2)	1	0.157
<5 servings of fruit and vegetable consumption/day	61 (95.3)	196 (98.4)	257 (97.7)		

*Physical activity*					
Less than the recommended level by the WHO (600 MET-minutes/week)	26 (40.6)	60 (30.1)	86 (32.6)	1	0.12
More than the recommended level by the WHO (600 MET-minutes/week)	38 (59.3)	139 (69.8)	177 (67.3)		

*BMI*					
Underweight	9 (14)	40 (20.1)	49 (18.6)	3	0.08
Normal	33 (51.5)	120 (60.3)	153 (58.9)		
Overweight	18 (28.1)	29 (14.5)	47 (17.8)		
Obese	4 (6)	10 (5)	14 (5.3)		

**Table 4:** Relationship between risk factor index and knowledge about hypertension.

Variables	Risk factor index			
	*r*	*p*	*M*	SD
Knowledge of reasons	−08	0.17	1.34	1.43
Knowledge of consequences	0.04	0.50	0.79	1.08
Knowledge of preventive measures	−11	0.06	1.38	1.48

**Table 5:** Binary logistic regression analysis shows association of risk factors and demographic variables with hypertension.

Variables	OR (95% CI)	*p*
Smoking behaviour	0.29 (0.09–.96)	0.04
Smokeless tobacco use	0.85 (0.42–1.70)	0.64
Fruit and vegetable consumption	1.32 (1.01–1.74)	0.04
Physical activity	0.55 (0.27–1.14)	0.11
Body mass index	1.85 (1.21–2.84)	0.01
Alcohol consumption	1.48 (0.55–3.97)	0.43
Gender	0.70 (0.32–1.52)	0.36
Education	0.93 (0.73–1.18)	0.56
Occupation	0.99 (0.87–1.13)	0.95
Age group	1.55 (1.14–2.11)	0.01

## Data Availability

The authors declare access to data is restricted due to confidentiality.
